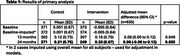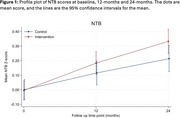# The APPLE Tree programme randomised controlled trial: Effect of Active Prevention in People at risk of dementia through Lifestyle, bEhaviour change and Technology to build REsiliEnce

**DOI:** 10.1002/alz70861_108516

**Published:** 2025-12-23

**Authors:** Harriet Demnitz‐King, Mariam M Adeleke, Julie Barber, Michaela Poppe, Jessica Budgett, Sweedal Alberts, Larisa Duffy, Hannah Chapman, Rosario Isabel Espinoza Jeraldo, Oliver Kelsey, Malvika Muralidhar, Sedigheh Zabihi, Elisa Aguirre, Nicholas Bass, Henry Brodaty, Alexandra Burton, Paul Higgs, Rachael M Hunter, Jonathan D Huntley, Helen C Kales, Iain A Lang, Natalie L Marchant, Sarah Morgan‐Trimmer, Anne‐Marie Minihane, Penny Rapaport, Miguel Rio, Irene Petersen, Karen Ritchie, Zuzana Walker, Kate Walters, Marina Palomo, Claudia Cooper

**Affiliations:** ^1^ Queen Mary University of London, London UK; ^2^ UCL, london UK; ^3^ University College London, London UK; ^4^ Queen Mary University of London, London, London UK; ^5^ North East London NHS Foundation Trust, Ilford UK; ^6^ University of New South Wales (UNSW), Sydney, NSW Australia; ^7^ University of Exeter, Exeter UK; ^8^ University of California, Davis, Sacramento, CA USA; ^9^ College of Medicine and Health, University of Exeter, Exeter UK; ^10^ University of East Anglia, Norwich UK; ^11^ INSERM, Montpellier France

## Abstract

**Background:**

Large‐scale trials of multidomain interventions indicate that modifying lifestyle and psychological risk factors can slow cognitive decline. This study evaluated whether a lower‐intensity, personally tailored secondary dementia prevention programme, informed by behaviour change theory, could reduce cognitive decline over two years in adults with subjective cognitive decline (SCD) or mild cognitive impairment (MCI).

**Method:**

We conducted a multi‐site, single‐blind randomised controlled trial (ISRCTN17325135) in England, recruiting 747 older adults with SCD or MCI between 05/10/2020 and 31/12/2022. Participants were randomised 1:1 to the 12‐month APPLE‐Tree intervention or control (usual care plus brief written dementia prevention information). The intervention focused on promoting healthy lifestyles, enhancing social connections and enjoyable activities, and improving self‐management of long‐term conditions. Shifting online due to COVID‐19, it comprised ten 1‐hour group video sessions over six months, delivered by two non‐clinical facilitators, supervised by a clinical psychologist. Sessions were supplemented with alternating video‐call “tea breaks” for informal interaction and biweekly individual goal‐setting calls. From months 6–12, participants continued with monthly “tea breaks”.

The primary outcome was cognition, measured by NTB composite score at 24 months. An intention‐to‐treat analysis was conducted using a three‐level mixed effects model including treatment arm, time, treatment‐by‐time interaction, baseline Neuropsychological Test Battery (NTB) score, and site as fixed effects. Random effects accounted for intervention arm group clustering and repeated measures at 12 and 24 months.

**Result:**

The trial recruited above target, with high intervention adherence (305/374 [82%] attending ≥5 main sessions). In the primary analysis that included 635/746 (85%) of participants randomised, the mean adjusted NTB scores increased over time in both arms (Table 1 and Figure 1), but the increment was higher in the intervention compared with the control arm (adjusted mean difference at 24 months: 0.06 [95% Confidence Interval ‐0.00–0.13], *p* =0.055).

**Conclusion:**

APPLE‐Tree provides an accessible, scalable model for secondary dementia prevention. The small effect size is comparable with previous successful, more intensive dementia prevention interventions with similar participant groups. Leveraging remote delivery and non‐clinical facilitators could enable wide‐scale implementation to support older adults with memory concerns. Secondary outcomes (e.g., quality of life, intervention‐targeted factors) available May 2025.